# Cytokines in sensitization to aeroallergens 

**DOI:** 10.5414/ALX1480E

**Published:** 2018-09-01

**Authors:** M. Albrecht, A.-M. Dittrich

**Affiliations:** Nachwuchsgruppe SFB 587; Pädiatrische Pneumologie, Allergologie und Neonatologie, Medizinische Hochschule Hannover

**Keywords:** asthma, sensitization, cytokines

## Abstract

Knowledge about the immunological mechanisms underlying asthma bronchiale is a prerequisite for development of new (causal) interventions. A large number of studies has proven asthma to be a complex disease with subtypes with different immunological features. Cytokines and chemokines, which are secreted by immune cells as well as structural cells play an important role not only in maintenance and amplification but have significant impact in the initiation of pulmonary inflammations – the asymptomatic sensitization phase. This article describes important immunological mediators in the context of the pulmonary sensitization phase. Moreover chances and constraints of intervention strategies aiming at these mediators are discussed. Several new aspects like classification of immunological phenotypes in bronchial asthma for individualized strategies and taking the sensitization phase into account, reveal possible targets among both “old acquaintances” like IL-4 and newly identified mediators (e.g. IL-17, IL-33).

**German version published in Allergologie, Vol. 35, No. 9/2012, pp. 446-453**

For decades, researches have been trying to elucidate the immunologic processes underlying bronchial asthma in order to find a starting point for causal therapy options. Shortly after Mosmann and Coffman [1] described the differentiation of CD4+ T helper cells (Th) into Th1 and Th2 subpopulations based on different cytokine patterns, M. Kapsenberg and colleagues used T cell clones from atopic and non-atopic patients to find that „atopic disorders of… (atopic donors) suffering from allergic asthma, result from the predominant occurrence of allergen-specific T cells that …. resemble murine Th2 cells…” [2]. This means that there is a relationship between bronchial asthma and the occurrence of Th2 cells, which mainly produce the cytokine interleukin (IL)-4 and only small amounts of interferon (IFN)-γ. 

In the following years, many research projects contributed to the knowledge of the complexity of the immunologic background of bronchial asthma and showed that the disease cannot be reduced to the presence of Th2 cells alone [3]. The pathogenesis of bronchial asthma involves numerous other cytokines, chemokines, and other cell types and their interrelationship has frequently been analyzed. So far, several therapeutics options have been developed based on the knowledge of the effector phase of bronchial asthma (anti-IgE, steroids, β_2_-mimetics, leukotriene receptor antagonists); however, they are mainly symptom-oriented and often expensive (e.g., anti-IgE). 

In order to find new options of intervention, it is necessary to also look at the asymptomatic sensitization phase that precedes the effector phase (where the first symptoms are observed) (Figure 1). In many cases, the severity of bronchial asthma correlates with the number of sensitizations present in a patient so that polysensitization is a risk factor for severe, difficult-to-treat asthma [4]. Thus, a better understanding of the sensitization phase could contribute to a better understanding of the immunological processes involved in the development of severe bronchial asthma. From this fact, new approaches for (causal) interventions could emerge which could possibly prevent polysensitizations and thus influence the chronification process of severe bronchial asthma. Furthermore, this type of investigation can contribute to the classification of “immunologic” phenotypes in order to evaluate if these different phenotypes respond better or worse to existing therapeutics and to develop individualized therapeutic strategies. Here, we are presenting immunological mediators of the sensitization phase (Figure 2) and discussing their potential as a possible target of intervention. 

## IL-4 

The early finding of the relationship between asthma and the occurrence of Th2 cells and their marker cytokine IL-4 is still a key feature of bronchial asthma and could also be confirmed on the genetic level in association studies on asthma and atopy [5]. IL-4 also plays a central role in the sensitization phase as this cytokine triggers the isotype shift of B cells towards the production of IgE antibodies and also is a factor of polarization for naïve T cells towards Th2. Studies in murine models have shown that the presence of IL-4 during antigen administration suffices to trigger sensitization against the previously unknown antigen without further immunological signals being necessary [6]. The source of this so-called “early” IL-4 is currently being investigated. It has been suggested that basophilic cells act as antigen-presenting cells and can induce a Th2-polarized immune response by simultaneous IL-4 secretion [7]. There might also be other cell types that produce “early” IL-4. Further functions of IL-4 in the lungs include activation of dendritic cells, stimulation of epithelial cells to produce mucus, recruiting of eosinophils, and activation of “alternatively activated” macrophages [8]. 

The central role of IL-4 in the initiation of asthma makes cytokines an interesting target for therapeutic interventions. 

Unfortunately, the use of a neutralizing antibody against IL-4 did not prove very effective in asthma therapy [9], and although the use of an IL-4 (and IL-13) receptor inhibitor during the effector phase showed promising results [10], the low number of study participants (24 – 32) reduces the validity of these results. 

One possible explanation for the limited effectiveness of anti-IL-4 therapy might be the high variety of functions of this cytokine which could be compensated by other mediators (e.g., IL-13). Thus, a combination therapy might perhaps be more promising. In addition, the question arises whether the use of IL-4 in certain immunological subtypes of the disease could show effects that have not yet been observed in studies in immunologically non-characterized patient cohorts. 

## TSLP 

The cytokine TSLP (thymic stromal lymphopoeitin) is secreted by epithelial cells, basophils, and mast cells and has an activating effect on mast cells and basophils. As TSLP also activates dendritic cells and enables them to polarize naïve T cells during antigen presentation to Th2 (by up-regulating OX40 ligand [11]), this cytokine plays a central role in the initiation of sensitizations. Studies in murine models demonstrate that different stimuli – among them some allergens – can induce production of TSLP by epithelial cells which then causes a Th2 immune response in the lungs via dendritic cells [12]. In experimental murine studies, the over-expression of TSLP led to an increased production of the Th2 cytokines IL-4, IL-5, and IL-13, and the animals subsequently expressed an asthmatic phenotype [13]. Another fact that could be demonstrated in the murine model is that the treatment with recombinant TSLP leads to a significant increase in IL-4-producing basophils in the blood which can induce a Th2-polarized immune response, as described above [14]. It has not fully been elucidated yet if the sensitization process in humans is similar. Association studies demonstrate a relationship between asthma and TSLP [15]. If TSLP plays a similar crucial role in the sensitization in humans, a well-directed intervention against this cytokine will be a promising approach for preventive asthma therapy; however, no clinical studies have been initiated yet. Current data from murine models demonstrate that blocking the TSLP receptor can prevent subsequent sensitization [16]. 

## IL-33 

Another recently described cytokine which is thought to have a role in Th2-polarized inflammation and allergy, is IL-33 that belongs to the IL-1 family. Interestingly, ST2, the receptor for IL-33, was identified much earlier as a marker for Th2 cells [17]. IL-33 is released from epithelial, endothelial, and mast cells, and the release can be triggered, e.g., by cell damage [18]. IL-33 contributes to the effector phase of the pulmonary inflammation by triggering the release of the cytokines IL-4, IL-5, and IL-13 in polarized Th2 cells and thus amplifies the Th2 response. In addition, in mice the treatment with IL-33 leads to the increased release of these cytokines as well as to the development of an asthmatic phenotype [19]. The fact that IL-33 can also contribute to the maturation and activation of dendritic cells and that, in the murine model, the transfer of dendritic cells treated with IL-33 leads to an increased eosinophil count and pulmonary mucus production shows that IL-33 also plays a role in the sensitization phase [20]. Several current genetic association studies demonstrated a relationship between IL-33, ST2, and asthmatic diseases so that an involvement of IL-33 in human bronchial asthma can be assumed [21]. Indeed, IL-33 also actives human basophils, eosinophils, and Th2 cells in vitro [22]. Because of the clear association of IL-33 and ST2 with asthmatic diseases, the potential of this relationship is currently being investigated as a target for successful intervention. Nevertheless, current experimental data are inconsistent: some studies demonstrated a rather regulatory function of ST2 in airway inflammation, while other publications describe an amplifying effect on the Th2-polarized pulmonary inflammation [18]. Thus, further investigation is necessary to evaluate IL-33 as a potential target in asthma therapy. Further studies should also clarify whether this cytokine plays a key role during the sensitization phase. 

## TNF-α 

The cytokine tumor necrosis factor alpha (TNF-α) is well known for its pro-inflammatory effects and shows pleiotropic effects in many immunological processes. It also plays a significant role in the asthmatic effector phase. Target cells include immune cells (T cells, macrophages, monocytes, eosinophils) as well as structural cells of the lung tissue (fibroblasts, smooth muscles, endothelial cells), on which TNF-α has various effects that can contribute to the development and amplification of airway inflammation. Murine experiments demonstrate that TNF-α can also influence the sensitization process. In these experiments, administration of TNF-α together with an antigen sufficed to induce pulmonary sensitization against the antigen without other immunological signals being present [23]. Also for this cytokine, human association studies have demonstrated a correlation between asthma and certain genetic polymorphisms [24], and high TNF-α levels in the blood could be related to severe forms of the disease and steroid insensitivity [25]. Several preparations for TNF-α inhibition have been tested in clinical studies, among them soluble TNF receptors, chimeric antibody constructions, and human monoclonal antibodies. First clinical studies using TNF-α antagonists were carried out in small patient cohorts and their results were promising [26]. A placebo-controlled follow-up study in a considerably larger number of patients (309) suffering from severe asthma was discontinued because of the high number of adverse events [27]. Among other things, the affected patients suffered from infections and various tumors – side effects making impossible the broad use of TNF-α antagonists for asthma therapy. 

## GM-CSF 

Granulocyte-monocyte colony-stimulating factor (GM-CSF) is secreted by activated T cells, macrophages, endothelial cells, and bone marrow cells and in its main function leads to the maturation and differentiation of myeloid cells, including dendritic cells. The presence of differentiated and activated dendritic cells is the basis for the antigen-specific immune response. Experiments have shown that GM-CSF is also able to mediate allergic sensitization. In these murine models, the over-expression of the factor together with intra-nasal antigen application led to the development of an eosinophilic airway inflammation [28]. Consequently, the neutralization of GM-CSF led to the reduction of symptoms and the Th2 response in another murine model of allergic airway inflammation [29]. The underlying mechanism of these effects in the induction of a Th2-polarized inflammation is that GM-CSF is a survival factor for eosinophils and Th2 cells. The role of GM-CSF in human asthma has been underlined by studies in which an increased GM-CSF level in the bronchoalveolar lavage was measured in asthma patients as compared to healthy controls after segmental challenge [30] as well as by studies on the genetic association in early-childhood asthma and other atopic diseases [31]. 

However, similar to TNF-α, GM-CSF has pleiotropic effects and its essential role in hematopoiesis does not allow for studies on the blockade of this cytokine in bronchial asthma. 

## IL-17 

The main member of the relatively newly discovered family of IL-17 cytokines is IL-17A. This cytokine is secreted by Th17 cells, CD8+ cells, γ-δ-T cells, and iNKT cells and has an important function in the immune defense against extracellular pathogens. Association studies also show a correlation of IL-17 with early-childhood asthma [32], and current investigation demonstrates a relationship between IL-17A and asthmatic diseases [33]. These studies investigated a subtype of bronchial asthma which is not characterized by eosinophilia but by a neutrophil-dominated airway inflammation. In patients suffering from this subtype, the number of neutrophils and the amount of neutrophil-recruiting IL-17 in various compartments correlated with the severity of the disease [34]. Additionally, there is evidence for the fact that this patient group is insensitive to steroid treatment [35]. 

Studies in murine models support the hypothesis that IL-17 and IL-17-producing Th17 cells play an important role in the resistance against standard therapeutic measures: the influx of neutrophils in the lungs which is triggered by IL-17 contributes to the resistance against glucocorticoid treatment [36]. Besides its neutrophil-recruiting activity, IL-17 also has effects on other types of cells, like endothelial, epithelial, dendritic, and muscle cells. All of these are activated and stimulated to produce pro-inflammatory molecules [37]. The fact that IL-17 plays a significant role in pulmonary sensitization processes could be demonstrated in a murine model in which an acute Th17-polarized airway inflammation sufficed to trigger sensitization against a previously unknown antigen [38]. Intra-nasal administration of a previously unknown antigen during the acute phase of the Th17-polarized inflammatory response in the lungs led to a new sensitization, which was, after a second challenge, characterized by lymphocytic influx into the bronchoalveolar lavage fluid, increased serum creatinine levels against the new antigen, and a clear airway hyperresponsiveness (Figure 3). Specific interventions against IL-17 are currently not being used in the therapy of asthma. First studies on the use of a monoclonal anti-IL-17 antibody in Crohn’s disease have been completed. Currently, there ongoing studies in further diseases like psoriasis, polymyalgia rheumatica, uveitis, and ankylosing spondilitis. The results of the completed studies have not been published so far. In principle, this antibody might be a good tool to treat the “neutrophil-dominated” steroid-resistant subtype of asthma in the future. 

## IL-25 

IL-25 (IL-17E) also belongs to the family of IL-17 cytokines, but is secreted by Th2 cells, basophils, eosinophils, endothelial cells, and mast cells. It also plays a role in the classical eosinophil/Th2-dominated type of asthma [39]. Experimental data derived from murine models show that IL-25 can initiate the induction of a Th2 response and of an asthmatic phenotype [40]. Reversely, the administration of IL-25-neutralizing antibodies prevents sensitization and airway hyperresponsiveness in the murine model of allergic airway inflammation [41]. Indeed, IL-25 expression is increased in the pulmonary tissue of asthma patients [42]. Furthermore, the results of a current experimental study suggest that the amplified effects of IL-25 during the pulmonary inflammatory response result from the indirect inhibition of IL-17A [43]. This relationship between IL-17A and IL-17E in airway inflammation needs to be further investigated in the future. 

Clinical studies have not been initiated so far, which is probably related to the fact that the mentioned research results are all relatively new. 

In the past years, various cytokines could be identified that play a central role in allergic sensitization and frequently also during the effector phase. Because of their side effect profile, some of them (TNF-α, GM-CSF) will surely not be promising candidates for a broad use in the treatment of bronchial asthma. Others, however, could well be an interesting treatment option for patients with certain subtypes of severe bronchial asthma, particularly if the eligible patients are subject to a thorough immunological characterization. Clinical studies on the topic will surely follow in the near future in order to verify the experimental evidence. The high number of recently identified mediators shows how complex the sensitization phase is, but also raises hopes that new strategies of intervention can be developed that would be able to cope with the disease’s complexity. 

**Figure 1.**
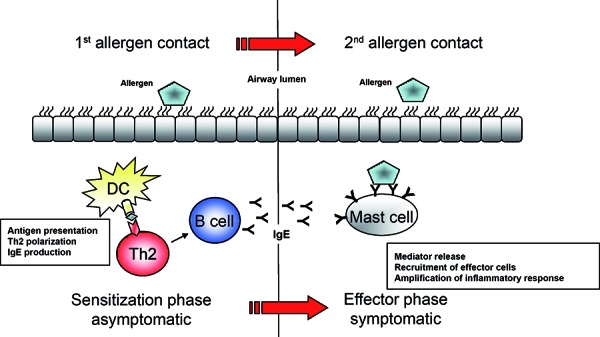
Sensitization phase and effector phase in allergic bronchial asthma. Th = T helper cell; DC = dendritic cell.

**Figure 2.**
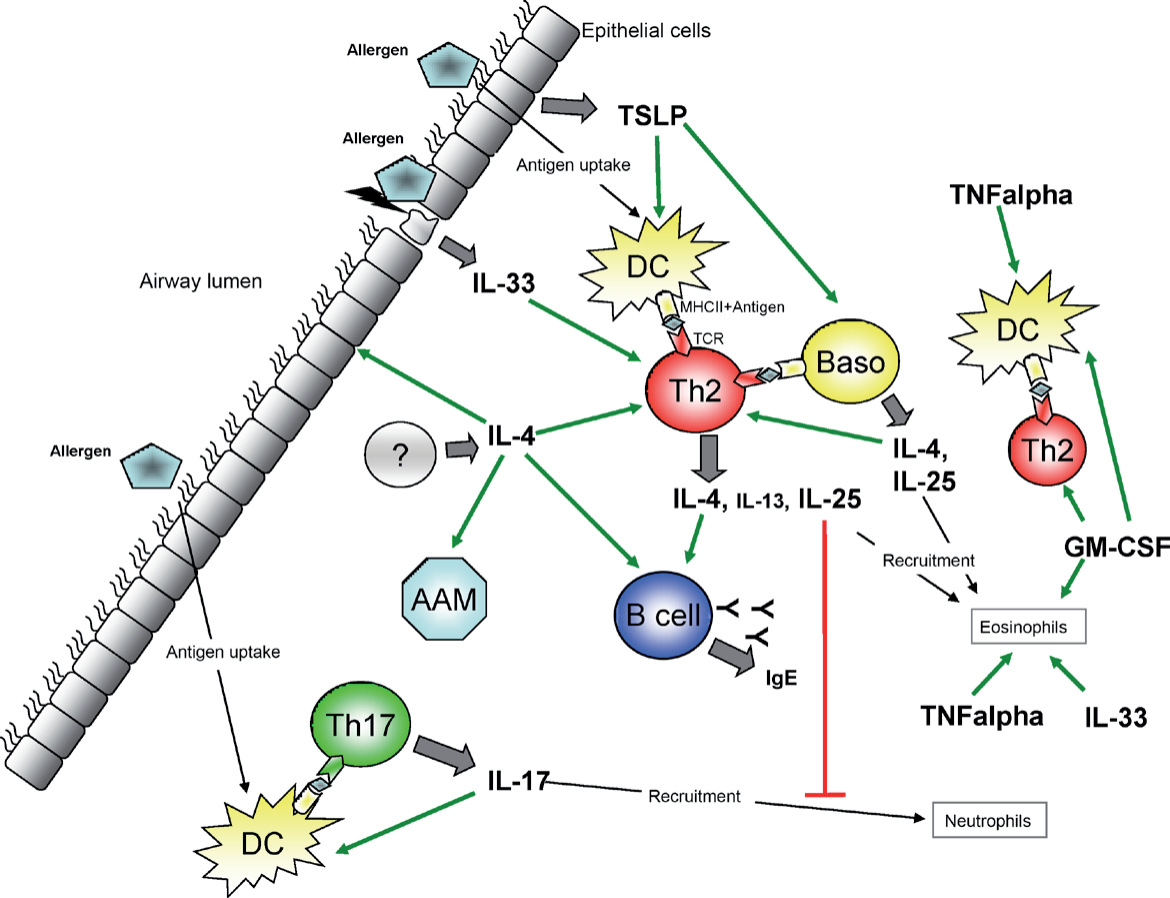
Schematic presentation of the cytokine network during pulmonary sensitization. For explanation, see text. Baso = basophil granulocyte; Th = T helper cell; DC = dendritic cell; AAM = alternatively activated macrophage; TSLP = thymic stromal lymphopoeitin; GM-CSF = granulocyte-macrophage-colony-stimulating factor; TNF = tumor necrosis factor; TCR = T cell receptor; MHCII = major histocompatibility complex II.

**Figure 3.**
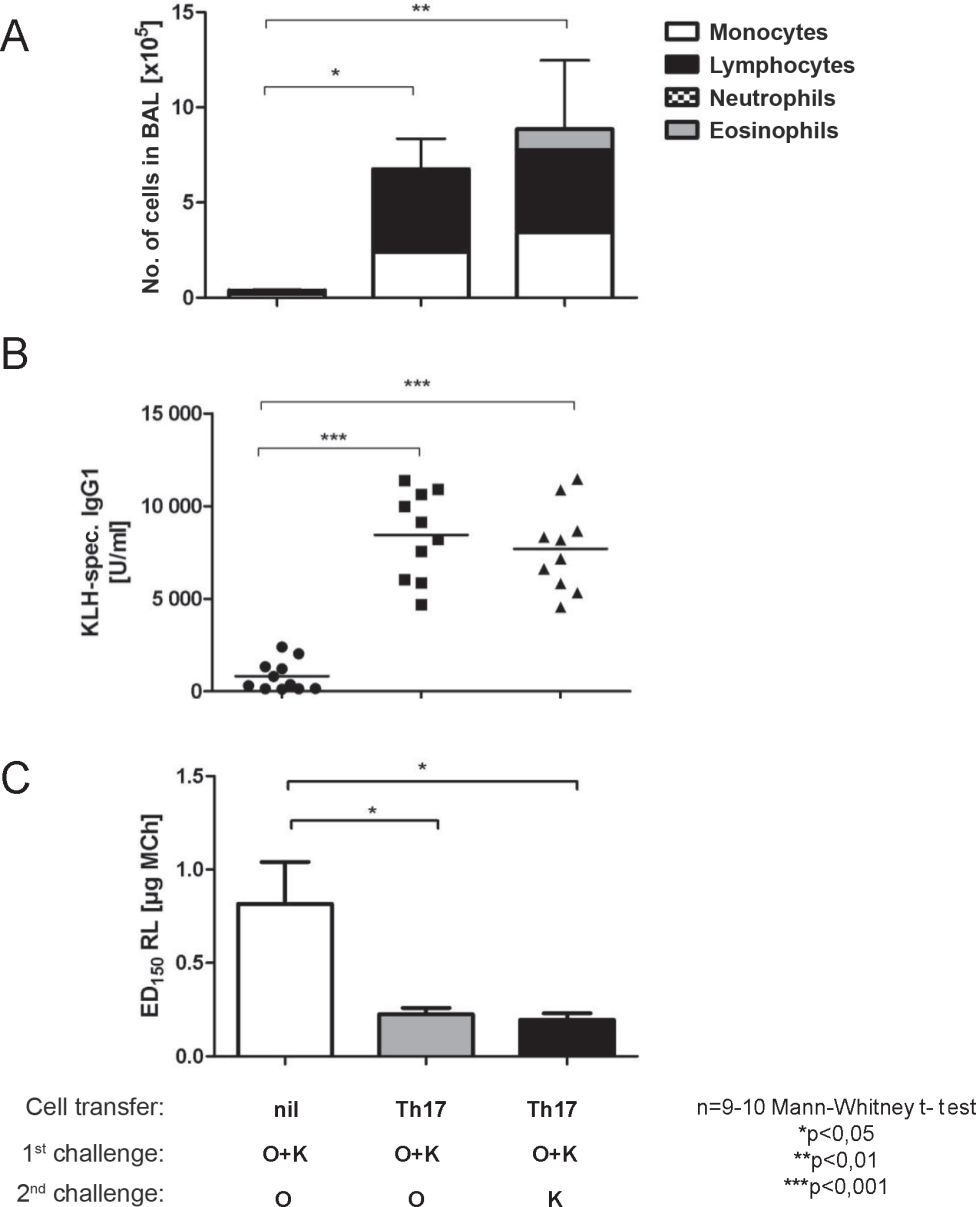
IL-17-producing Th17 cells mediate pulmonary new sensitization. After transferring ovalbumin-specific Th17 cells into naïve animals, the acute Th17-polarized airway inflammation is triggered by intra-nasal challenge (1^st^ challenge) with ovalbumin (O). When an unknown antigen (K) is simultaneously administered intra-nasally, new sensitization against this antigen occurs. This new sensitization is then confirmed in a 2^nd^ challenge phase by single administration of the previously unknown antigen (K) as compared to a positive control with the known antigen ovalbumin (O) (control animals do not receive cell transfer: nil): A: Airway inflammation: number and differentiation of cells in the bronchoalveolar lavage fluid (BAL). B: Systemic sensitization: KLH-specific antibody concentration in the serum. C: Airway hyperresponsiveness: effective methacholine dose (ED) necessary to achieve an increase in lung resistance (RL) by 150%.
